# Multispectral camouflage for infrared, visible, lasers and microwave with radiative cooling

**DOI:** 10.1038/s41467-021-22051-0

**Published:** 2021-03-22

**Authors:** Huanzheng Zhu, Qiang Li, Chenning Tao, Yu Hong, Ziquan Xu, Weidong Shen, Sandeep Kaur, Pintu Ghosh, Min Qiu

**Affiliations:** 1 State Key Laboratory of Modern Optical Instrumentation, College of Optical Science and Engineering, Zhejiang University, Hangzhou, China; 2 Key Laboratory of 3D Micro/Nano Fabrication and Characterization of Zhejiang Province, School of Engineering, Westlake University, Hangzhou, China; 3 Institute of Advanced Technology, Westlake Institute for Advanced Study, Hangzhou, China

**Keywords:** Mid-infrared photonics, Nanophotonics and plasmonics

## Abstract

Interminable surveillance and reconnaissance through various sophisticated multispectral detectors present threats to military equipment and manpower. However, a combination of detectors operating in different wavelength bands (from hundreds of nanometers to centimeters) and based on different principles raises challenges to the conventional single-band camouflage devices. In this paper, multispectral camouflage is demonstrated for the visible, mid-infrared (MIR, 3–5 and 8–14 μm), lasers (1.55 and 10.6 μm) and microwave (8–12 GHz) bands with simultaneous efficient radiative cooling in the non-atmospheric window (5–8 μm). The device for multispectral camouflage consists of a ZnS/Ge multilayer for wavelength selective emission and a Cu-ITO-Cu metasurface for microwave absorption. In comparison with conventional broadband low emittance material (Cr), the IR camouflage performance of this device manifests 8.4/5.9 °C reduction of inner/surface temperature, and 53.4/13.0% IR signal decrease in mid/long wavelength IR bands, at 2500 W ∙ m^−2^ input power density. Furthermore, we reveal that the natural convection in the atmosphere can be enhanced by radiation in the non-atmospheric window, which increases the total cooling power from 136 W ∙ m^−2^ to 252 W ∙ m^−2^ at 150 °C surface temperature. This work may introduce the opportunities for multispectral manipulation, infrared signal processing, thermal management, and energy-efficient applications.

## Abstract (web summary)

Manipulating electromagnetic waves to camouflage objects is an important tool. Here, the authors present a camouflage that covers a wide range of frequencies based on multilayer and metasurface technologies.

## Introduction

Nature is the best inspiration for camouflage, which is extensively adopted by various species such as cephalopods, chameleons, and other animals to disguise themselves against predators^1–4^. In modern military and civil applications, camouflage technology aims to conceal the signature of objects and render them invisible from the potential threats^5^. Conventionally, camouflage is only confined in a particular spectral range, e.g., visible^3,6^, mid-infrared (MIR)^7–13^, or microwave^14^. However, a combination of advanced detectors operating in different wavelength bands (e.g., visible to MWIR camera^15^, SAR, and IR combination^16^) developed in recent years entails a compatible camouflage among different spectral bands, i.e., multispectral camouflage^17–21^.

Manipulating electromagnetic waves for multispectral camouflage is a challenging task due to different camouflage principles in different bands and the necessity to cover the wide spectrum range of wavelength from hundreds of nanometers (visible) to centimeters (microwave)^15–17^. To satisfy the requirements of multispectral camouflage, the spectral properties of the device should be: (i) low emittance in the MIR atmospheric windows i.e., mid-wavelength infrared (MWIR) 3–5 μm and long-wavelength infrared (LWIR) 8–14 μm against thermal imagers and heat-seeking missiles^19–26^; (ii) characteristic reflection in the visible (380–780 nm) range for background matching^27,28^; (iii) high absorbance in the microwave radar band (X-band, 8–12 GHz) for reducing radar cross-section (RCS)^29^ and (iv) high absorbance at the laser wavelengths (1.55 μm and 10.6 μm)^30,31^. According to the Stefan-Boltzmann law, the thermal radiation intensity is proportional to the fourth power of the surface temperature; therefore, reduction of the surface temperature is desired for IR camouflage^32–35^, especially for the military objectives with high surface temperature (e.g., converging nozzles of aircraft (~950 K)^36^, tailpipe of aircraft (~740 K)^37^, and funnels of naval ships (~680 K)^38^). However, radiative heat transfer is blocked in traditional MIR camouflage with broadband low emittance, introducing heat instability^39,40^. In addition, camouflage against active detection systems including microwave and lidar further aggravates the heat instability due to absorbed microwave/laser energy^18^. To mitigate the severe heat instability and reduce surface temperature, high emittance is required in the MIR non-atmospheric window 5–8 μm for radiative cooling and wavelength-selective emitters have been proposed^18,41–45^. Although the MIR compatible multispectral camouflage including visible^27,46,47^, microwave^18,48,49^, or laser^30,31^ bands have been demonstrated, there is no existing material that simultaneously satisfies all the aforementioned requirements. Consequently, advanced technology for multispectral camouflage and radiative cooling still needs to be explored to ensure multispectral compatible functionality and effectiveness.

In this paper, we demonstrate multispectral camouflage for MIR, visible, laser, and microwave bands with efficient radiative cooling in the non-atmospheric window. By taking advantage of the wavelength difference of the spectral bands, the device realizes visible, MIR, and laser camouflage, as well as radiative cooling by the Ge/ZnS multilayer wavelength-selective emitter, and the microwave camouflage by the Cu-ITO-Cu absorptive metasurface. This structure presents several advantageous characteristics: (1) low emittance of 0.11/0.12 in the MWIR/LWIR bands for MIR camouflage, and high emittance of 0.61 in the non-atmospheric window for radiative cooling; (2) multiple visible colors for color matching in different backgrounds (e.g., soil, desert, water body, and vegetation); (3) high absorbance (>0.9) in X-band and (4) high absorbance at 1.55 μm and 10.6 μm lasers wavelengths. Compared with broadband low emittance material Cr, the wavelength selective emitter shows a decrease of 8.4/5.9 °C for inner/surface temperature and reduction of 53.4/13.0% for IR signal, with a heat source of 2500 W ∙ m^−2^ power density. Furthermore, comparison experiments between vacuum and normal pressure reveal that the natural convection can be enhanced by radiation in the non-atmospheric window, and therefore the cooling power is enhanced to improve MIR camouflage performance.

## Results

### Multispectral camouflage

**Fig. 1:**
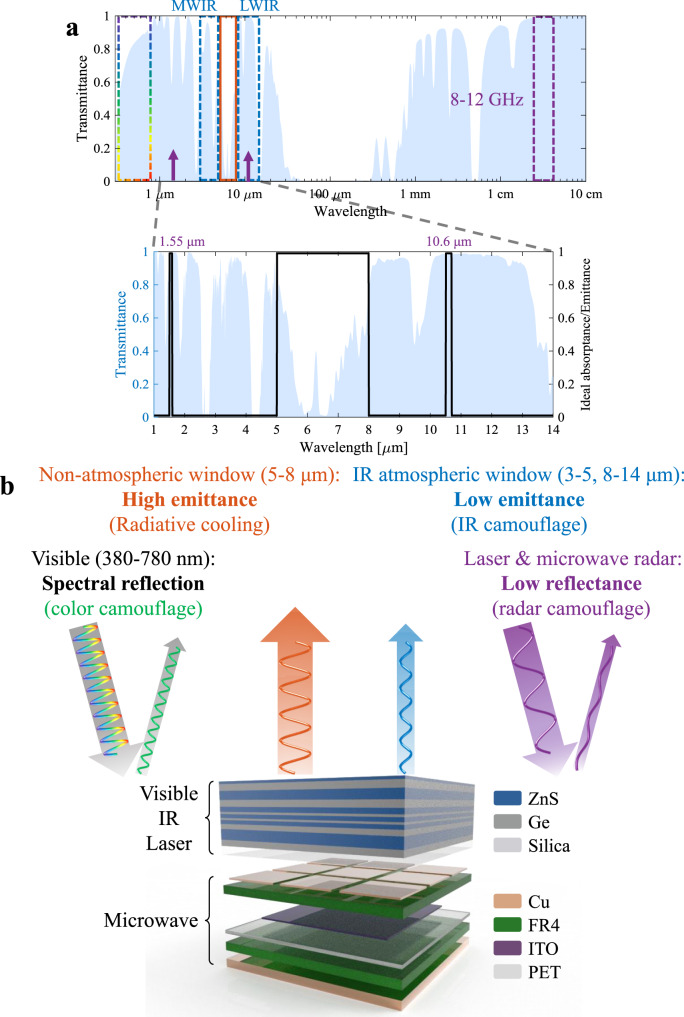
Atmosphere transmittance and scheme for multispectral camouflage. **a** Atmosphere transmittance spectrum for the electromagnetic wave (from visible to microwave) with detectable ranges indicated by dashed boxes and arrows. The IR part (1–14 μm) is magnified and shown with the ideal absorbance/emittance spectrum. **b** Requirements of multispectral camouflage for each respective range, and the designed structure with ZnS/Ge multilayer selective emitter for visible, IR, and laser camouflage and Cu-ITO-Cu absorptive metasurface for microwave camouflage.

Multispectral camouflage depends on the detection principles in different wavelength bands, and the detectability of the wavelength spectrum (dashed boxes and short arrows in Fig. 1a) is closely related to the transmittance of electromagnetic waves of that wavelength through the atmosphere (light blue shaded area in Fig. 1a). In the MIR atmospheric window (blue dashed boxes), low emittance (blue arrow in Fig. 1b) is required as most thermal imagers and heat-seeking missiles operate in this wavelength region^15^, whereas in the non-atmospheric window (5–8 μm, orange box in Fig. 1a), the thermal radiation intensity of an object with a moderate temperature, (100–300 °C) reaches the maximum, thus high emittance is required for radiative cooling without influencing MIR camouflage (orange arrow in Fig. 1b). At the typical lidar’s laser wavelengths, e.g., 1.55 μm and 10.6 μm (purple short arrows in Fig. 1a), high absorbance is required for reducing the reflection or other back-scattering. On the other hand, high absorbance at 10.6 μm is in conflict with the low emittance requirement in the LWIR band, hence requiring a sufficiently narrow bandwidth for a 10.6 μm absorption peak (Fig. 1a). For the visible range (380–780 nm, colored dashed box in Fig. 1a), the reflectance spectrum is required to be close to the background to form the resembling color as the background. For the microwave range (8–12 GHz, X-band), high absorbance is demanded low reflectance or scattering (purple arrows in Fig. 1b), which is similar to the lidar.

To overcome the challenge of all spectral requirements with one structure, the combined structure is fabricated with different layers to satisfy corresponding spectral requirements. The spectral compatibility is ensured by leveraging the wavelength difference. The combined structure (Fig. 1b) consists of a Ge/ZnS multilayer wavelength-selective emitter (SE) for visible/lasers/MIR camouflage with radiative cooling and a Cu-ITO-Cu microwave absorptive metasurface.

**Fig. 2:**
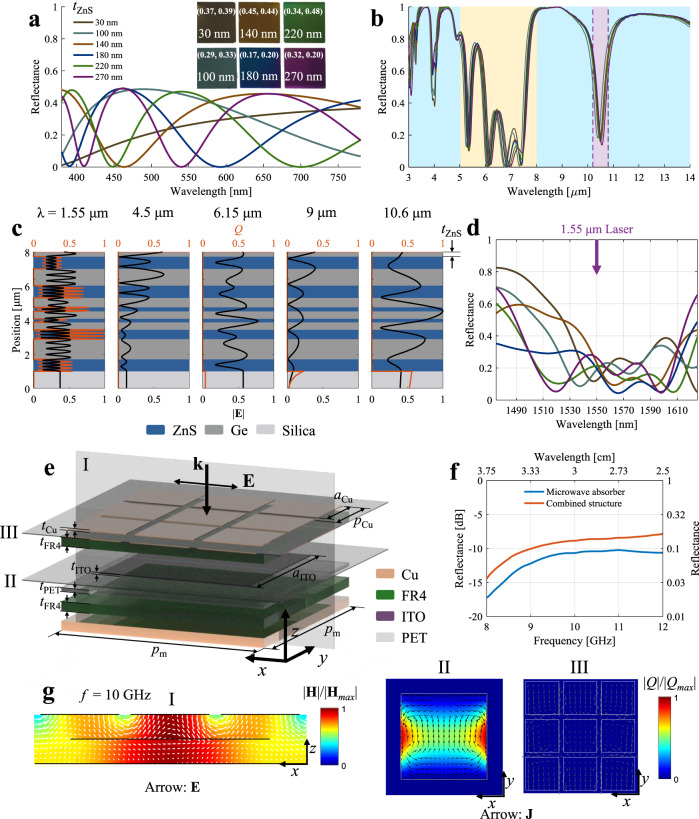
Multispectral camouflage for visible, MIR, lasers, and microwave. **a** The measured visible reflectance and optical images for top ZnS layer of thickness varying from 30 nm to 270 nm. The chromaticity coordinates (*x*, *y*) are written on the corresponding optical images (insets). **b** The measured reflectance in the MIR range for the different values of top ZnS layer thickness. **c** The electric field |**E**| and resistive loss *Q* in the ZnS/Ge multilayer selective emitter for wavelengths of 1.55 μm, 4.5 μm, 6.15 μm, 9 μm, and 10.6 μm. The thicknesses of the Ge/ZnS multilayer films are 0.721/0.982/0.721/0.559/0.234/0.438/0.206/0.438/0.552/1.18/0.701 μm (from top to bottom). **d** The reflectance spectrum near 1.55 μm. **e** The Cu-ITO-Cu microwave absorptive metasurface. **f** The reflectance spectra corresponding to the Cu-ITO-Cu microwave absorber metasurface and the combined structure. **g** The normalized magnetic field intensity (color) and electric field (arrow) on the cross-section I (*x*–*z* plane, parallel to polarization); the normalized resistive loss (color) and surface currents (arrow) on the cross-sections II and III, respectively (*x*–*y* plane, perpendicular to wave vector). The frequency is 10 GHz.

For visible range, the spectrum is determined only by the thickness (*t*_ZnS_ in Fig. 2c) of the top ZnS layer as the beneath Ge layer (0.72 μm) is opaque. By varying the *t*_ZnS_ from 30 nm to 270 nm, anti-reflection peaks are formed at different wavelengths. Fig. 2a demonstrates the experimental reflectance for different *t*_ZnS_ and the corresponding colors with the optical image in the inset with CIE-1931 chromaticity coordinates (*x*, *y*). The thickness of the top ZnS layer can be chosen to achieve background matching colors, e.g., 30/140 nm for soil/deserts background, 180 nm for clear water body background, and 220 nm for green vegetation background^50^.

For IR range, high reflectance (low emittance) in the MWIR and LWIR bands along with low reflectance (high emittance) in the non-atmospheric window is realized with all ZnS/Ge multilayers. The thicknesses are optimized by the genetic algorithm, with the optimization targets of maximum absorbance in the non-atmospheric window, maximum absorbance at 10.6 μm and 1.55 μm wavelengths, and minimum absorbance in both atmospheric windows. The measured band emittance at 100 °C for MWIR/LWIR is 0.11/0.12, and band emittance for non-atmospheric window (5–8 μm) is 0.61 (see Supplementary note 1 for band emittance definition). The MIR spectra of the SE are hardly influenced by the top ZnS layer, due to its negligible thickness compared to wavelength (Fig. 2b, see Section S3 for SE reflectance spectra for different *t*_ZnS_). Apart from the top ZnS layer, the thicknesses of the Ge/ZnS (gray/blue blocks) multilayer films from top to bottom are optimized as shown in Fig. 2c. The Ge/ZnS layers form a one-dimensional photonic crystal with forbidden bands located at MWIR and LWIR ranges, as shown by the electric field (|**E**|) and resistive heat loss (*Q*) distribution at 4.5 μm and 9 μm wavelengths (Fig. 2c). For the non-atmospheric window outside the forbidden bands, the electric field does not decay within the Ge/ZnS layers, forming high absorbance within the lossy silica substrate.

For lidar’s laser wavelength, the narrowband low reflectance at 10.6 μm wavelength is realized with the fifth to seventh Ge/ZnS layers (top to bottom, excluding the top ZnS layer), as shown by the electric field distribution at 10.6 μm in the Fig. 2c. The forbidden band of the one-dimensional photonic crystal in LWIR is broken by these three layers, where high electric field intensity is formed. For another lidar’s laser wavelength of 1.55 μm, the absorbance is mainly realized by the intrinsic loss within the Ge layers (extinction coefficient *k* = 0.0056 at 1.55 μm). The measured reflectance value at 1.55 μm wavelength for various *t*_ZnS_ is less than 0.3, and therefore camouflage for 1.55 μm lidar is achieved.

For the microwave X-band, low reflectance is observed for the Cu-ITO-Cu microwave absorber (the structure shown in Fig. 2e), which is characterized by a return loss lower than −10 dB, and the corresponding absorbance is higher than 90% (Fig. 2f). The periodic array of Cu square blocks with thickness *t*_Cu_ = 0.18 μm, array period *p*_Cu_ = 3.67 mm, and block side length *a*_Cu_ = 3.2 mm on flame retardant-4 (FR4, thickness *t*_FR4_ = 1 mm) form a frequency selective surface for the X-band. With the Cu substrate, the magnetic field is enhanced between the frequency selective surface and reflective substrate (Fig. 2g). With the electrically lossy ITO layer of thickness *t*_ITO_ = 175 nm, side length *a*_ITO_ = 8.1 mm, and sheet resistance 35 Ω ∙ sq^−1^ on 175-μm-thick PET, the enhanced magnetic field, therefore, introduces high ohmic loss (Fig. 2g). Together with the selective emitter part, the return loss is still lower than −8.25 dB for the combined structure, corresponding to the absorbance higher than 85%. Therefore, the combined structure is compatible with MIR, visible, laser, and microwave camouflage, as well as radiative cooling.

### IR camouflage demonstration

**Fig. 3:**
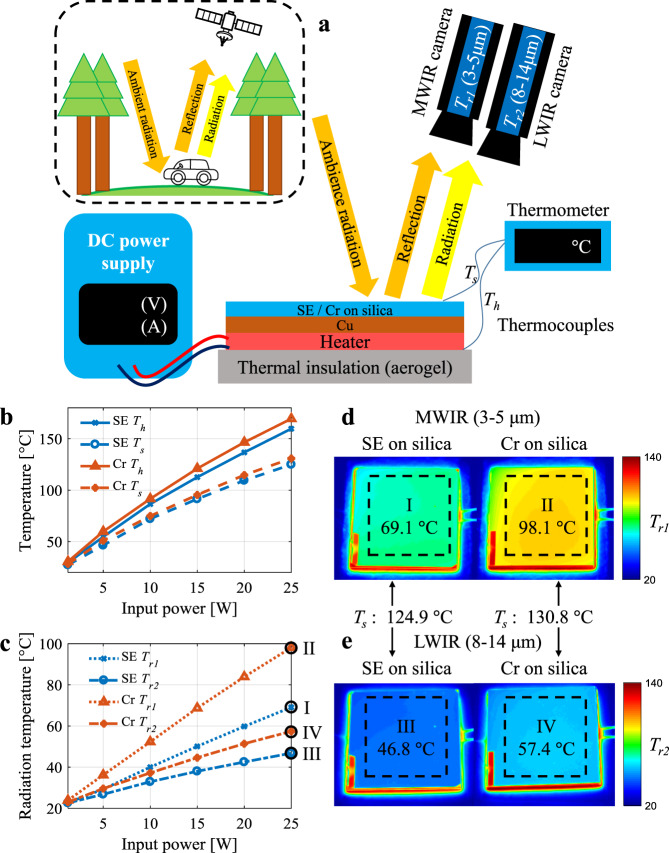
IR camouflage demonstration. **a** Schematic diagram of the experimental configuration for IR camouflage and radiative cooling performance demonstration. **b** Measured heater/surface temperature *T*_*h*_/*T*_*s*_ for SE and Cr at different input heating power values. **c** Measured radiation temperature *T*_*r1*_/*T*_*r2*_ in MWIR/LWIR band for SE and Cr at different input power values. **d** IR images of SE and Cr at 25 W input power captured by MWIR band thermal imager. **e** IR images of SE and Cr at 25 W captured by LWIR band thermal imager.

The camouflage for the MIR range is complicated due to spectral conflicts among MIR camouflage, radiative cooling, and laser camouflage. To demonstrate the performance of MIR camouflage, the SE part of the multispectral camouflage structure is compared with the Cr film on the same silica substrate as a conventional broadband low emittance surface, in terms of absolute temperature and radiative temperature reduction. For both SE and Cr cases, the constant power input is applied to the heater beneath the samples (Fig. 3a). The silica aerogel plate under the heater is used for thermal insulation, and the copper plate ensures horizontal temperature uniformity. The heater temperature *T*_*h*_ and sample surface temperature *T*_*s*_ are measured with the input power varying from 1.25 W to 25 W (Fig. 3b). Both the heater temperature *T*_*h*_ and the surface temperature *T*_*s*_ of the SE are lower than those of Cr for the same input power values, and the temperature difference between SE and Cr increases with the input power. At 25 W input power, *T*_*h*_/*T*_*s*_ of SE is about 8.4/5.9 °C lower than that of Cr, indicating efficient dissipation for internal/surface heat through radiative cooling.

The radiative (apparent) temperature *T*_*r1*_ and *T*_*r2*_ in MWIR and LWIR range, respectively, are measured for quantitatively evaluating the IR camouflage performance (Fig. 3c). Under the indoor configuration, both the reflected ambient radiation and radiation from the sample are collected by the thermal imagers, which is analogous to the scene of a forest or city with considerable ambient radiation shown in the dashed box in Fig. 3a. The radiative temperature of SE is significantly lower than Cr, due to lower surface temperature *T*_*s*_ and lower band emittance (see Supplementary note 9). At 25 W input, the surface temperatures *T*_*s*_ are 124.9 °C and 130.8 °C for SE and Cr, respectively. In the MWIR band, the radiation temperatures *T*_*r1*_ of SE (region I) and Cr (region II) are reduced to 69.1 °C and 98.1 °C (Fig. 3c), respectively, and in the LWIR band, the radiation temperature *T*_*r2*_ of SE and Cr are reduced to 46.8 °C and 57.4 °C, respectively. Compared to Cr, the SE shows a 53.4% (3.32 dB) reduction of IR signal intensity in the MWIR range (band integrated radiation intensity for SE/Cr is 22.6/48.6 W ∙ m^−2^) and a 13.0% (0.606 dB) reduction of radiation intensity in LWIR range (band integrated radiation intensity for SE/Cr is 229/264 W ∙ m^−2^). The SE can endure a temperature of at least 300 °C (see Supplementary note 10), which ensures its applicability for objects with high temperatures.

### Enhanced natural convection by radiation

**Fig. 4:**
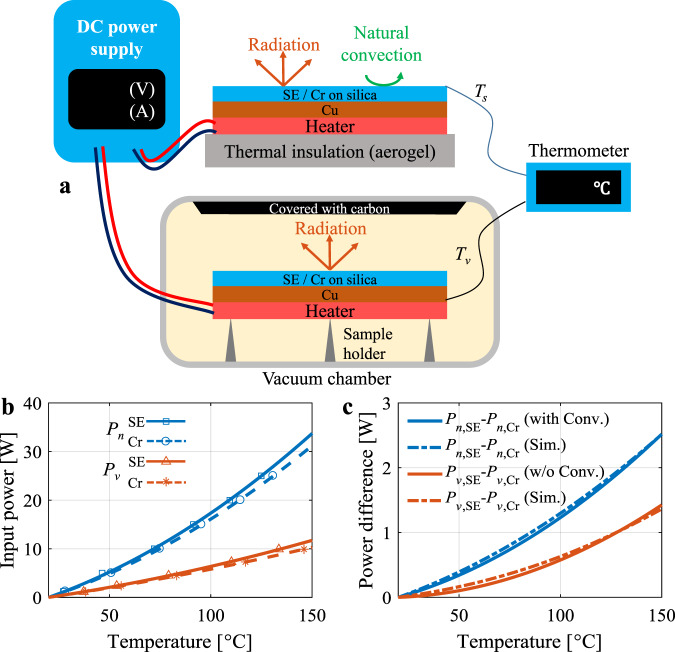
Validation experiments of enhanced natural convection by radiation. **a** Schematic diagram showing a comparison of experimental setups between normal pressure and vacuum conditions. **b** The required input power for steady-state temperatures under normal pressure (*P*_*n*_) and vacuum (*P*_*v*_) conditions. **c** The input power difference between the SE and Cr at two conditions (with/without natural convection for normal pressure/vacuum) and simulated power differences with/without natural convection.

Under normal temperature and pressure, natural convection dominates heat transfer for stationary objects. Natural convection is detrimental in sub-ambient radiative cooling and should be avoided^51–53^; whereas for IR camouflage, as the radiative cooling operates at a temperature higher than ambiance for heat dissipation, the natural convection is beneficial to improve the cooling capacity by coupling to radiation. In the non-atmospheric window (5–8 μm), the atmosphere is opaque mainly due to the absorption of the radiation power in the water vapor present in the ambient atmosphere; therefore, the radiation power is received by the water vapor in the ambient atmosphere. Enhancement of natural convection by radiation in the non-atmospheric window is demonstrated by the comparison experiments between the normal pressure condition and the vacuum condition (Fig. 4a). In the normal pressure condition with a relative humidity of 60%, both the natural convection and radiation are available for cooling of the sample under electric heating; whereas in the vacuum condition with a pressure of ~1 Pa, the natural convection is excluded. Other heat transfer channels, including conduction, side convection, and wire convection/radiation are regarded as parasitic loss, which are identical for SE and Cr at the same temperature.

The temperature is measured for different steady-state input power values for SE and Cr in both the normal pressure condition and the vacuum condition (Fig. 4b). To ensure the identity of radiative power between the normal pressure condition and the vacuum condition, the carbon surface is cooled with forced convection by air, and its temperature is close to ambient temperature, with the temperature monitoring for both sides (inside and outside the vacuum cavity). To reveal the influence of radiation on convection, the power differences between SE and Cr for both conditions are shown in Fig. 4c. If there is no coupling between radiation and convection, the power difference between SE and Cr should remain the same for both pressure conditions. Here, the power difference for the normal pressure condition (with convection) is larger than that of the vacuum condition (without convection), indicating enhanced natural convection due to coupled radiation. At the surface temperature of 150 °C, the power difference for normal pressure conditions is 2.52 W, which is larger than that of vacuum conditions (1.36 W), corresponding to an increased power density difference to 252 W ∙ m^−2^ from 136 W ∙ m^−2^ (sample area of 0.01 m^2^). For both with/without convection, the experimental power differences (solid lines) are consistent with the simulations (dash-dotted lines, see Supplementary note 9). The natural convection in the atmosphere can be enhanced with radiation in the non-atmospheric window, which provides a new approach for increasing the cooling power of devices when forced convection and radiative cooling in the atmospheric window are not available. This is especially significant for IR camouflage in which the radiation in the atmospheric window is strictly forbidden.

## Discussion

Multispectral camouflage for visible, IR, laser, and microwave ranges is demonstrated together with efficient radiative cooling through the multilayer wavelength-selective emitter and the microwave metasurface. First, multispectral camouflage is realized with the design based on wavelength difference among MIR, visible, laser, and microwave ranges, whose spectral compatibility is ensured by the layer-wise functionality. Second, the utilization of SE not only resolves the spectral conflict between IR camouflage and radiative cooling, as well as laser camouflage but also enhances the performance of IR camouflage due to excellent radiative cooling. Third, it is revealed that the radiation in the non-atmospheric window can further enhance the natural convection and therefore increase the cooling power, which is significant in applications when other heat transfer channels are restricted. Last, the SE based on ZnS/Ge multilayer films and the microwave metasurface based on common PCB processes are easy to fabricate and therefore hold potential for large-area applications. Ultimately, this work may open the opportunities for simultaneous manipulation of multispectral electromagnetic waves and heat transfer, thus paving the way towards military and civil applications, thermal management, and energy-efficient devices^54–61^.

## Methods

### Selective emitter fabrication

The ZnS/Ge multilayer film was deposited using the E-beam evaporation technique on a silica substrate with deposition rates of 1.5 nm/s (ZnS) and 0.5 nm/s (Ge).

### Optical characterization

The reflectance spectrum in the visible/NIR band was measured by a spectrophotometer (Cary7000, Agilent). The reflectance in the MIR band was measured by the FTIR microscope (Hyperion 1000, Brucker) with an FTIR spectrometer (Vertex 70, Brucker) through an MCT detector. The reflectance in the microwave band was measured by a vector network analyzer (8722C, Keysight) with a sample size of 200 mm × 200 mm.

### Radiation/absolute temperature measurements

The radiation temperature was measured with an LWIR camera (Blackbird precision IR-OEM-Module, Jenoptik) with a detection wavelength range of 8–14 μm and an MWIR camera (FAST M200, Telops) with a detection wavelength range of 3–5 μm. The absolute temperature was measured by thermocouples (5TC-TT-K-30-36, Omega) at normal pressure and thermal resistors (Pt100, TZ-CMEI) in the vacuum with a thermometer (THTZ408R, Tenghui, Ningbo).

### Simulations

The heat transfer simulation was performed with a heat transfer module and surface-to-surface radiation module in COMSOL Multiphysics. The microwave metasurface was designed with a radio frequency module (electromagnetic waves, frequency domain) in COMSOL Multiphysics. The electric field distribution is also simulated with a radio frequency module (electromagnetic waves, frequency domain) in COMSOL Multiphysics (the relative permittivity of Ge/ZnS are available in Supplementary note 2).

## Supplementary information

Supplementary Information

## Data Availability

The data that support the plots within this paper and other findings of this study are available from the corresponding author upon reasonable request.

## Code availability

Codes used for this work are available from the corresponding author upon reasonable request.
