# Supplementary material for: Multispectral camouflage for infrared, visible, lasers and microwave with radiative cooling
Source: Nat Commun. 2021 Mar 22;12:1805. doi: 10.1038/s41467-021-22051-0 (PMC7985314; doi:10.1038/s41467-021-22051-0)
Supplement: Supplementary file 1 — Supplementary Information [file 41467_2021_22051_MOESM1_ESM.pdf]

Supplementary Information

Multispectral camouflage for infrared, visible, laser and microwave with radiative cooling

Huanzheng Zhu[1], Qiang Li[1, a)], Chenning Tao[1], Yu Hong[1], Ziquan Xu[1], Weidong Shen[1], Sandeep Kaur[1], Pintu Ghosh[1] and Min Qiu[2, 3, b)]

[1]*State Key Laboratory of Modern Optical Instrumentation, College of Optical Science and Engineering, Zhejiang University, Hangzhou 310027, China.*

[2]*Key Laboratory of 3D Micro/Nano Fabrication and Characterization of Zhejiang Province, School of Engineering, Westlake University, 18 Shilongshan Road, Hangzhou 310024, China.*

[3]*Institute of Advanced Technology, Westlake Institute for Advanced Study, 18 Shilongshan Road, Hangzhou 310024, China.*

a) E-mail: qiangli@zju.edu.cn

b) E-mail: qiumin@westlake.edu.cn

Supplementary note 1. Band emittance

The band emittance of the selective emitter is calculated with the following formula:

$$\varepsilon_{\lambda_1-\lambda_2}=\frac{\int_{\lambda_1}^{\lambda_2}\varepsilon(\lambda)M_{bb}(\lambda,T)\,\mathrm{d}\lambda}{\int_{\lambda_1}^{\lambda_2}M_{bb}(\lambda,T)\,\mathrm{d}\lambda} \tag{1}$$

Supplementary note 2. Design of ZnS/Ge multilayer film selective emitter (SE)

The thickness of ZnS/Ge multilayer SE is determined by a genetic algorithm, with the following optimization targets - maximum absorbance in the non-atmospheric window, maximum absorbance at 10.6 μm and 1.55 μm, and minimum absorbance in both atmospheric windows. To be specific, the figure of merit is determined as:

$$\begin{gathered}FOM_{band}=\frac{\int_{3\mu m\text{ or }8\mu m}^{5\mu m\text{ or }14\mu m}R(\lambda)M_{bb}(\lambda,T)\,\mathrm{d}\lambda}{\int_{3\mu m\text{ or }8\mu m}^{5\mu m\text{ or }14\mu m}M_{bb}(\lambda,T)\,\mathrm{d}\lambda}+\frac{\int_{5\mu m}^{8\mu m}\left(1-R(\lambda)\right)M_{bb}(\lambda,T)\,\mathrm{d}\lambda}{\int_{5\mu m}^{8\mu m}M_{bb}(\lambda,T)\,\mathrm{d}\lambda}\\ FOM=\begin{cases}FOM_{band},\ R(10.6\mu m)\le 0.1\,\&\,R(1.55\mu m)\le 0.1\\ 0.5FOM_{band},\ R(10.6\mu m)\le 0.3\,\&\,R(1.55\mu m)\le 0.3\\ 0,\ R(10.6\mu m)>0.3\,|\,R(1.55\mu m)>0.3\end{cases}\end{gathered} \tag{2}$$

where the FOM_{band} evaluates the band integrated targets, and *FOM* evaluates the total camouflage and radiative cooling performance considering threshold values at two laser wavelengths. In optimization, the genetic algorithm is applied to search the largest value of *FOM* by varying the thickness of ZnS/Ge multilayers.

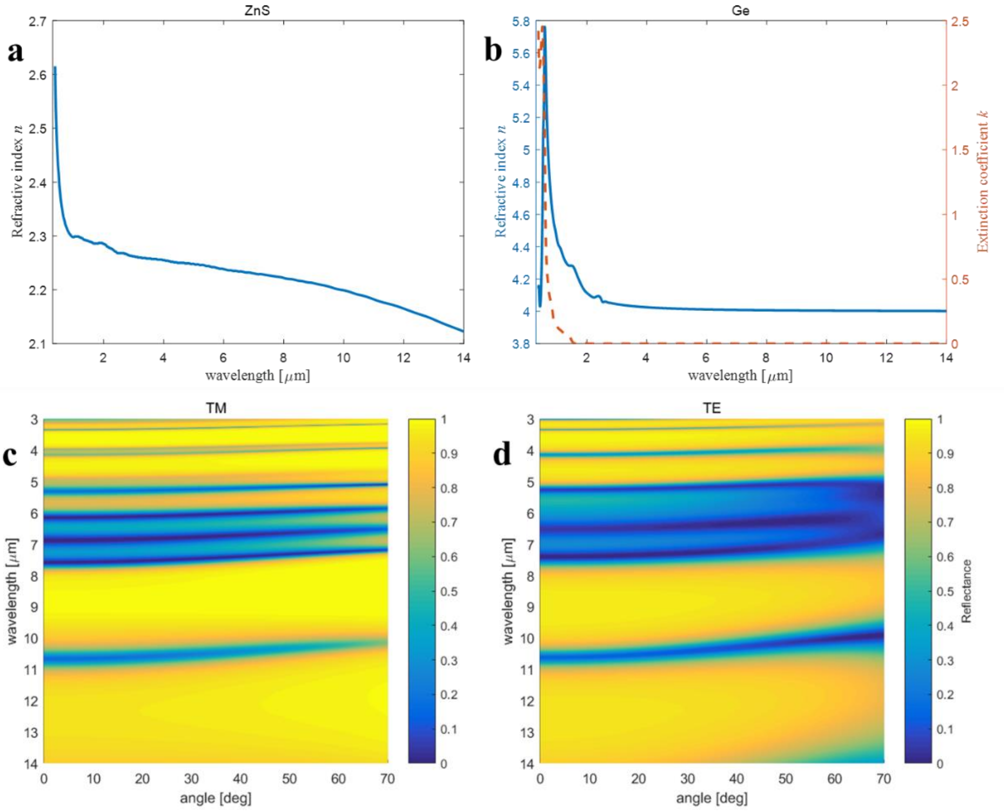

Supplementary Figure 1. The optical constants and angle dependence of SE. (a) and (b) Refractive

index n and extinction coefficient k for ZnS and Ge. (c) and (d) Simulated angle-dependent reflectance of the SE for both polarizations.

Supplementary note 3. Reflectance spectrum variation with different t_{ZnS}

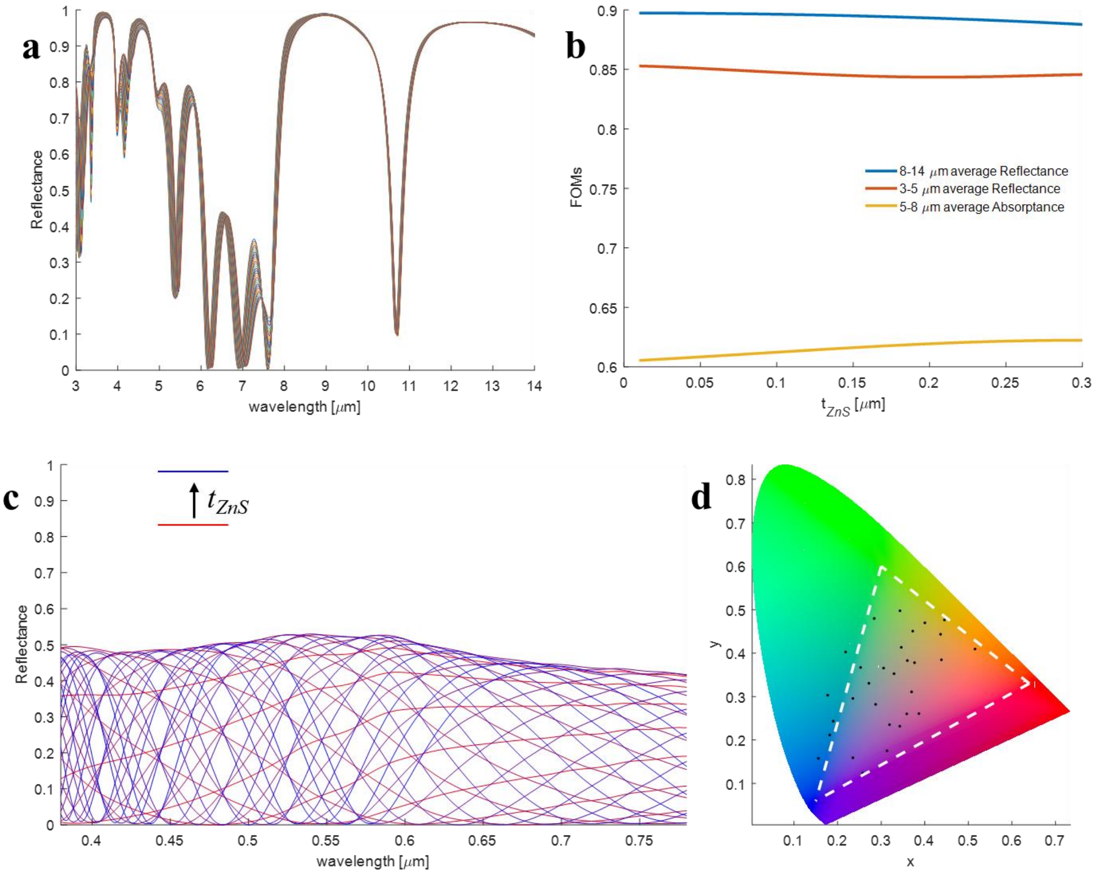

Supplementary Figure 2. Reflectance spectrum variation with different t_{ZnS}. (a) MIR reflectance spectra for different t_{ZnS} varying from 10 nm to 300 nm. (b) The figure of merit plots, which are band average reflectance for 3-5 and 8-14 μm or band average absorbance for 5-8 μm (Higher the value, better it is). (c) Visible reflectance spectrum for different t_{ZnS} varying from 10 nm to 300 nm (10 nm intervals). (d) The corresponding visible colors of spectra in (c) indicated in the CIE 1931 chromaticity diagram (Black dots) and the sRGB gamut (White dashed triangle).

Supplementary note 4. Angle dependence of reflectance spectra for visible, NIR, MIR, microwave bands

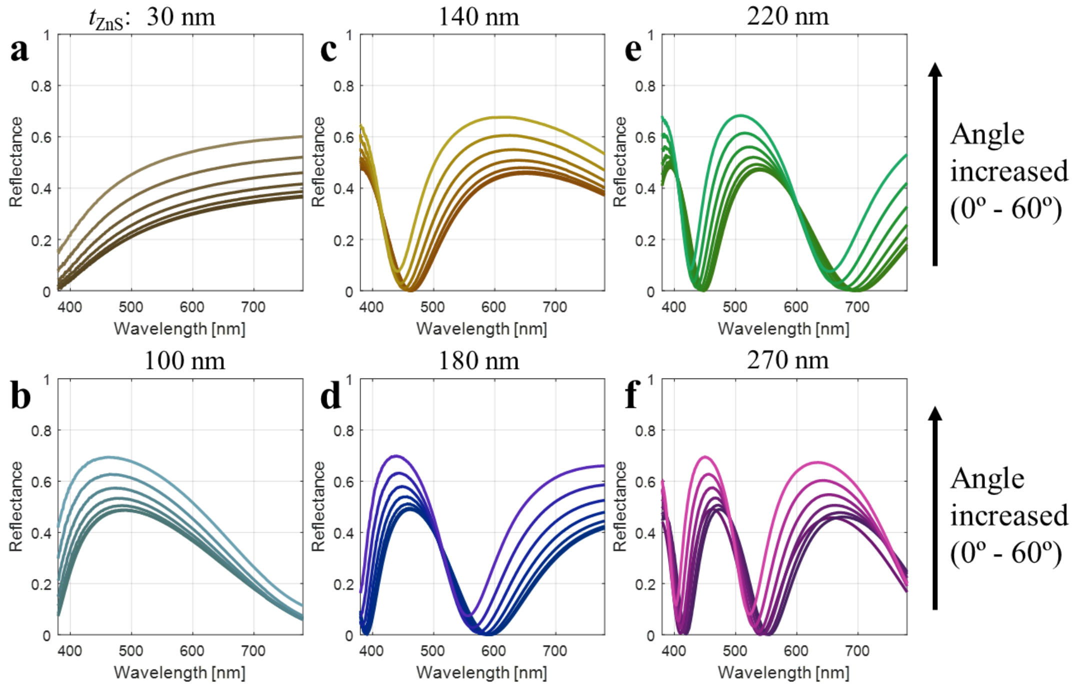

Supplementary Figure 3. Angle dependence (incident and detection angle: 0° - 60°, with an interval of 10°) of reflectance spectra in the visible range for different top ZnS layer thickness of (a) 30 nm, (b) 100 nm, (c) 140 nm, (d) 180 nm, (e) 220 nm, and (f) 270 nm. The reflectance curves are shown with the corresponding visible colors.

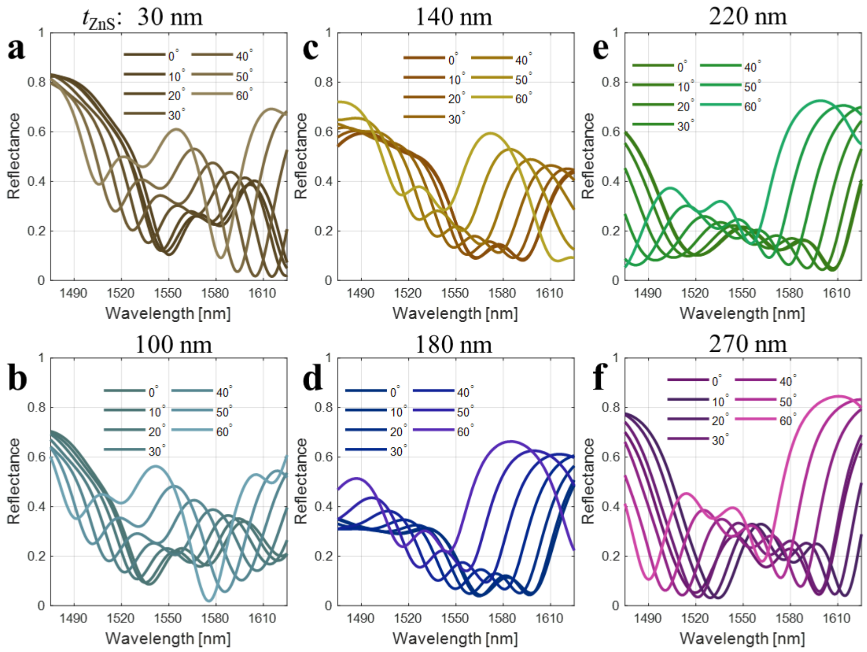

Supplementary Figure 4. Angle dependence (incident and detection angle: 0° - 60°, with an interval of 10°) of reflectance spectra near 1550 nm for different top ZnS layer thickness of (a) 30 nm, (b) 100 nm, (c) 140 nm, (d) 180 nm, (e) 220 nm, and (f) 270 nm.

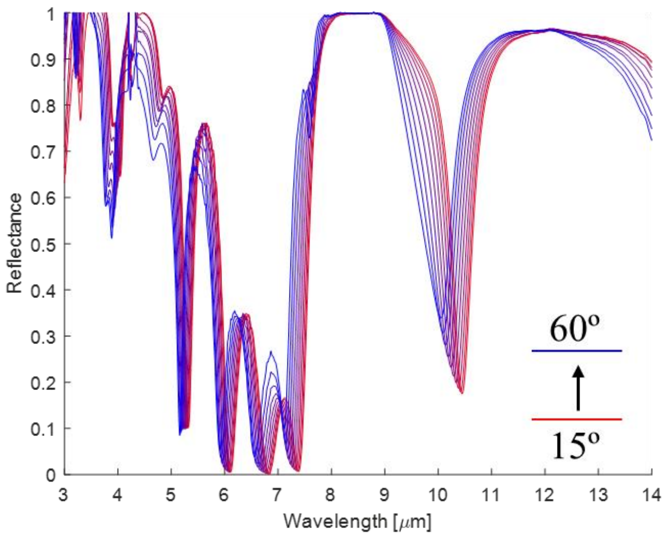

Supplementary Figure 5. Angle dependence (incident and detection angle: 15 ° - 60 °, with an interval of 5 °) of reflectance spectra in the MIR range.

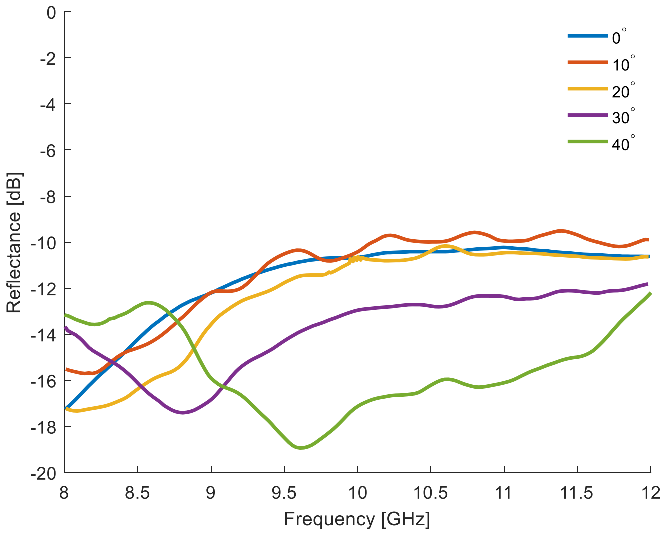

Supplementary Figure 6. Angle dependence (incident and detection angle: 0 ° - 40 °, with an interval of 10 °) of reflectance spectra in microwave X-band.

Supplementary note 5. Infrared images measured at different viewing angles

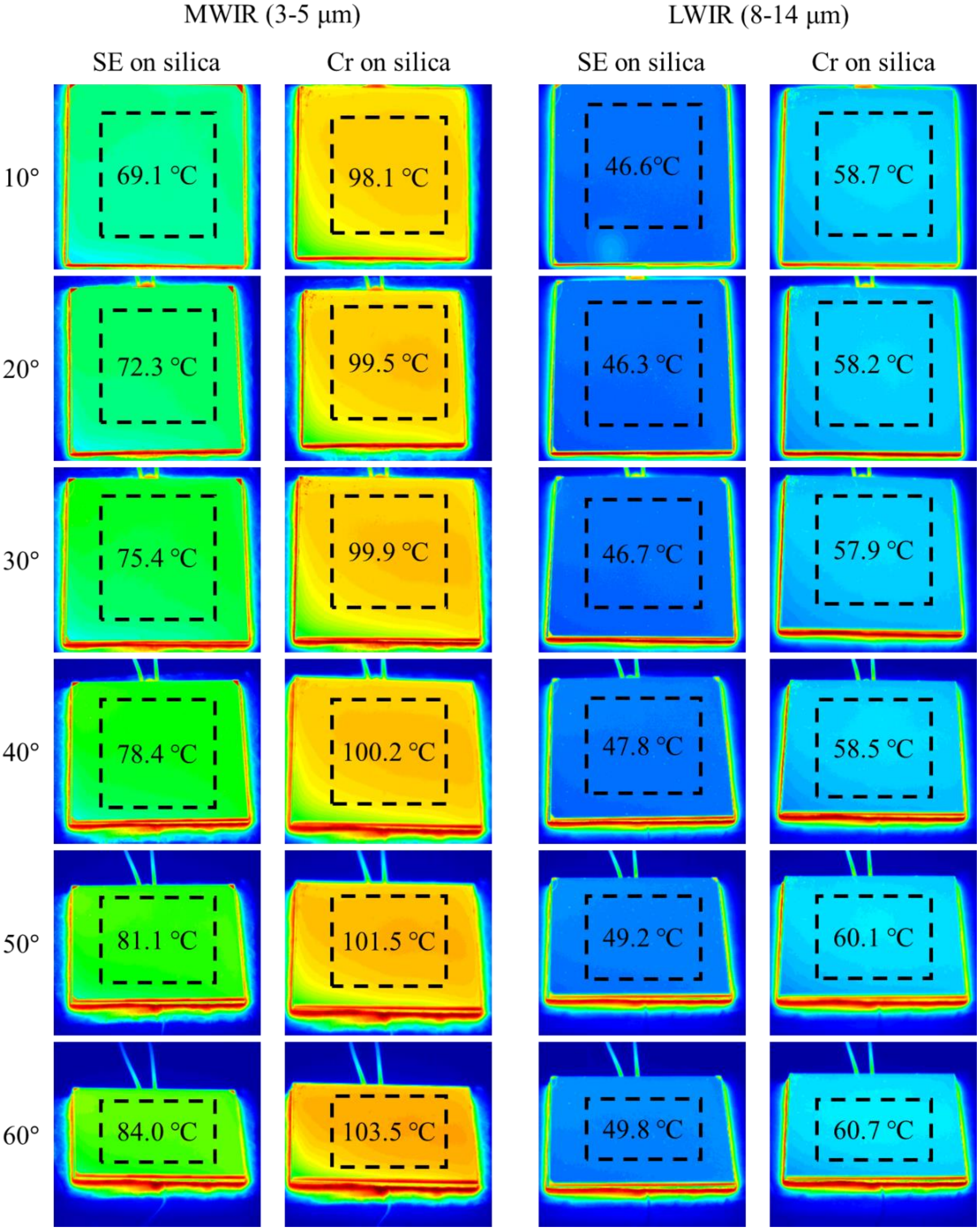

Supplementary Figure 7. The infrared images measured at different viewing angles (10 °- 60 °) of the thermal imagers for both MWIR and LWIR bands of SE and Cr samples.

Supplementary note 6. Measured absorbance and emittance of SE and Cr film in IR camouflage demonstration

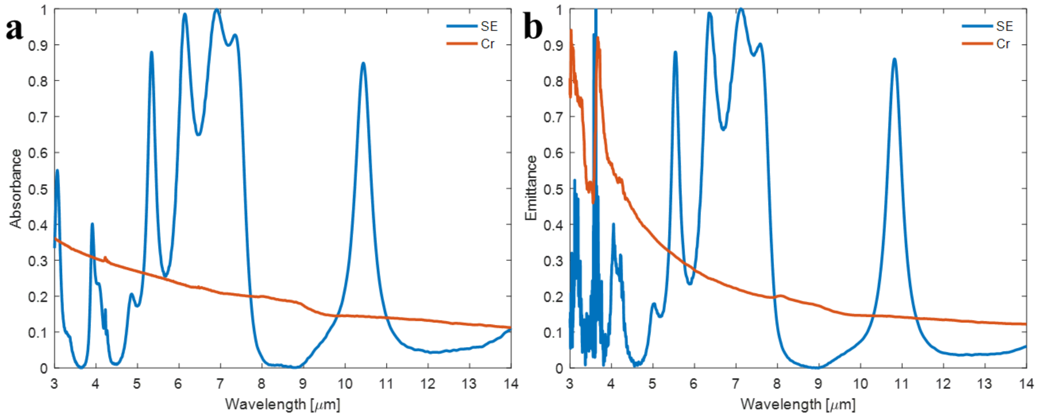

Supplementary Figure 8. Measured (a) absorbance and (b) emittance spectra for SE and Cr. The emittance is measured at 300 ℃, and therefore the emittance for wavelength shorter than 4 μm is noisy.

Supplementary note 7. Radiation temperature distribution

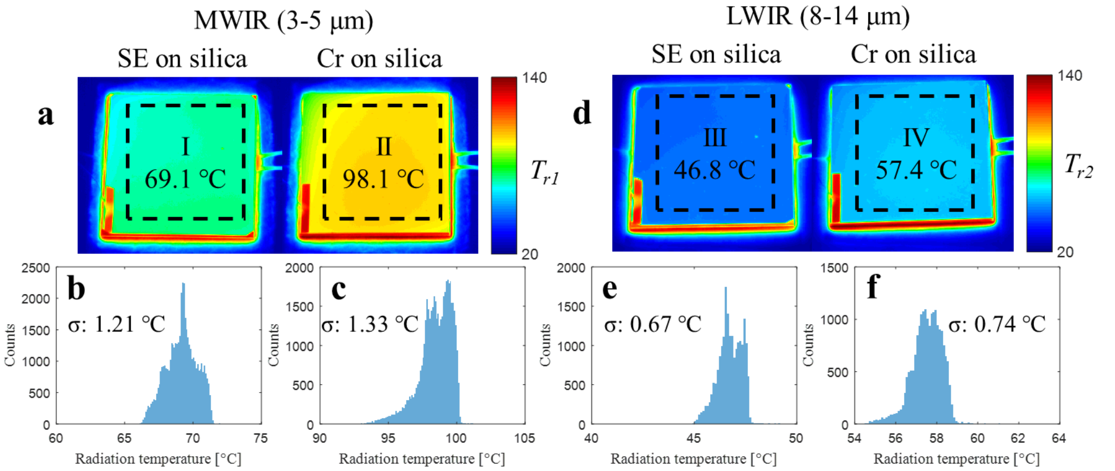

Supplementary Figure 9. Radiation temperature distribution. (a) IR images of SE and Cr at 25 W input power captured by MWIR band thermal imager, and the histograms for radiation temperature of (b) SE and (c) Cr. (d) IR images of SE and Cr at 25 W captured by LWIR band thermal imager, and the histograms for radiation temperature of (e) SE and (f) Cr.

Supplementary note 8. Demonstration on the flexible substrate

For the large and curved surface applications, the devices are required to be flexible to fit the surface shape. The SE part and the microwave absorber part are fabricated on the flexible substrate, as shown in Supplementary Figure 10. For the Se part, the multilayer is deposited on the polyimide (PI) substrate with a thickness of 0.2 mm, which guarantees its flexibility on potentially applicable objects. For the microwave part, the top Cu patterns are fabricated with the flexible printed circuit (FPC) also on the PI substrate. With the ITO pattern already on the flexible PET substrate, the microwave absorber can also be employed on a curved surface.

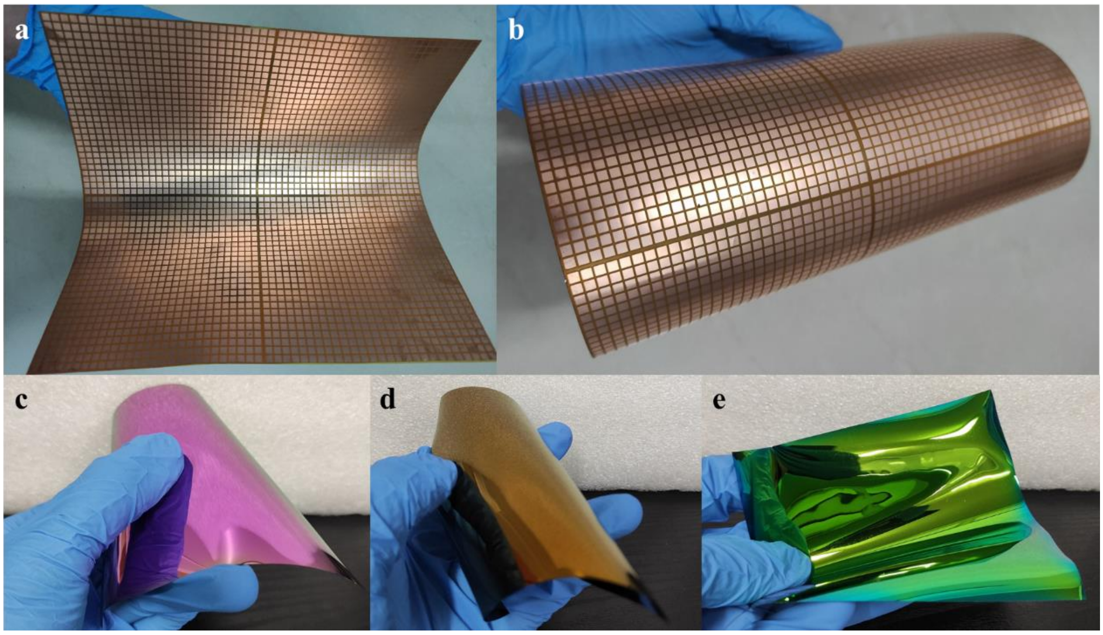

Supplementary Figure 10. Demonstration of devices on the flexible substrate for (a-b) the microwave absorber, and (c-e) the SE part.

Supplementary note 9. Heat transfer modeling

For normal pressure case, the power input at thermal equilibrium state (stable state) for different temperature can be defined as

$$q_n(T_s) = h_{conv}(T_s - T_{amb}) + \int_{3\mu m}^{14\mu m} \left(M(\lambda, T_s) - \varepsilon_{amb}(\lambda) M(\lambda, T_{amb})\right) \varepsilon(\lambda) \mathrm{d}\lambda + h_{para}(T_s - T_{amb})$$
$$M(\lambda, T) = \frac{2\pi hc^2}{\lambda^5 \left(\exp\left(\frac{hc}{k_B \lambda T}\right) - 1\right)} \tag{3}$$

where h_{conv} is the convective heat transfer coefficient, $M(\lambda, T)$ is the spectral radiance of blackbody, $\varepsilon(\lambda)$ is the spectral emittance, and h_{para} denotes heat transfer coefficient for other all heat transfer channels.

For vacuum case, the power input can be defined as

$$q_v(T_s) = \int_{3\mu m}^{14\mu m} \left(M(\lambda, T_s) - \varepsilon_{amb}(\lambda) M(\lambda, T_{amb})\right) \varepsilon(\lambda) \mathrm{d}\lambda + h_{para}(T_s - T_{amb}) \tag{4}$$

As it is difficult to determine the value of temperature-related h_{para} due to heat dissipation by conduction and radiation/convection on the sides of the structure, the difference between power inputs for SE and Cr under identical experiment configuration eliminates the influence of parasitic heat loss by considering the emittance of ambiance to be 1 constantly:

$$\Delta q_n(T_s) = \left(h_{conv,SE} - h_{conv,Cr}\right)(T_s - T_{amb}) + \int_{3\mu m}^{14\mu m} \left(M(\lambda, T_s) - M(\lambda, T_{amb})\right)\left(\varepsilon_{SE}(\lambda) - \varepsilon_{Cr}(\lambda)\right) \mathrm{d}\lambda$$
$$\Delta q_v(T_s) = \int_{3\mu m}^{14\mu m} \left(M(\lambda, T_s) - M(\lambda, T_{amb})\right)\left(\varepsilon_{SE}(\lambda) - \varepsilon_{Cr}(\lambda)\right) \mathrm{d}\lambda \tag{5}$$

Therefore the $\Delta q_v(T_s)$ can be determined by the surface temperature and emittance of samples, as shown by the simulated power difference without convection (vacuum condition, $P_{v,\mathrm{SE}}$-$P_{v,\mathrm{Cr}}$) in Fig. 4(c).

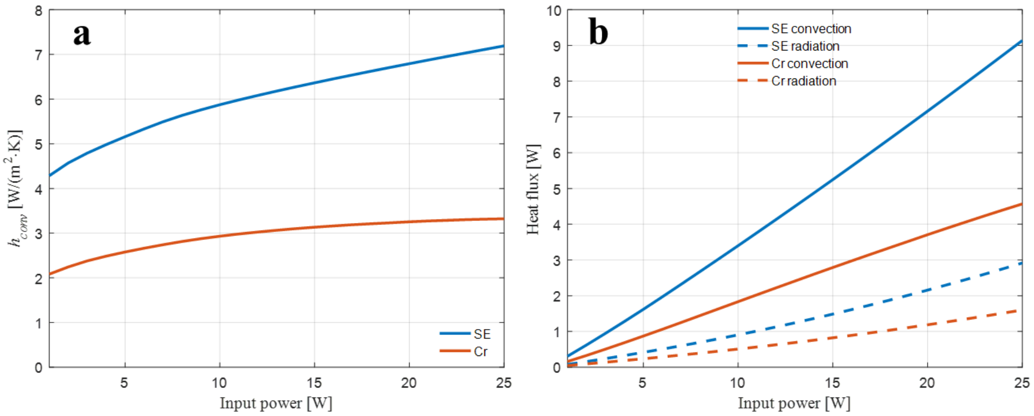

Supplementary Figure 11. Heat transfer coefficients. (a) The convective heat transfer coefficients h_{conv} on the samples' surfaces versus input powers. (b) The convective heat flux and radiative heat flux in the surface normal directions.

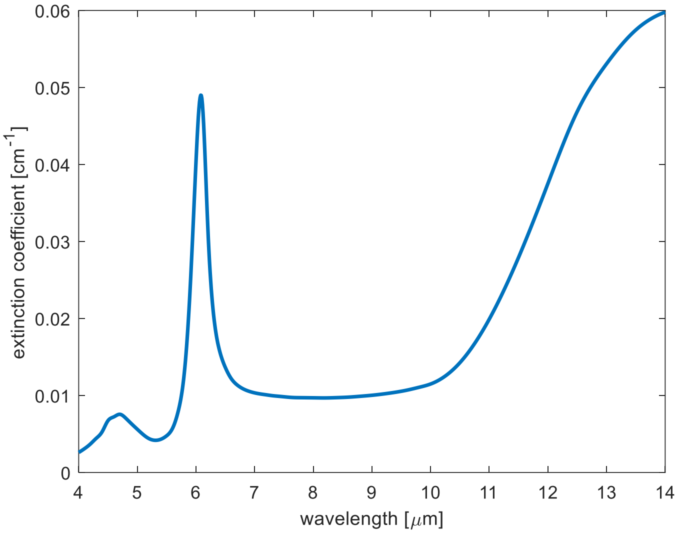

Supplementary Figure 12. The spectrum of the extinction coefficient of the atmosphere at infrared wavelengths.

For the $\Delta q_n(T_s)$, the convective heat transfer coefficients are dependent on the surface temperature, therefore related to the input power. The h_{conv} for both SE and Cr cases are obtained by *conjugate heat transfer* (coupling to *laminar flow* for natural convection) simulations in COMSOL Multiphysics, as shown in Supplementary Figure 11(a). For the Cr case, as the surface emittance is relatively low in the MIR band, the radiative heat transfer is simplified as *surface-to-ambient radiation* with a constant surface emittance 0.2. For the SE case, the wavelength-selective emittance is simulated with temperature-dependent surface emittance and the heat transfer is modelled with coupling among *heat transfer in solids*, *radiation in participating media*, and *laminar flow*. The absorption coefficient of the atmosphere is determined by band average of the extinction coefficients k of water vapor and relative humidity identical to experiments' value (60 %), the spectrum of the extinction coefficient of the atmosphere at infrared wavelengths is shown in Supplementary Figure 12. In the *radiation in participating media* module, the P1 approximation is applied for radiation

discretization. The mesh independence is indicated by similar results (velocity, pressure, and temperature field) between 1.7 mm maximum element size and 2.4 mm maximum element size for the fluid part.

By coupling to radiation, the convection of SE is enhanced indicated by both larger h_{conv} and convective heat flux compared to the Cr, as shown in Supplementary Figure 11. The difference in radiation heat flux between SE and Cr is mainly related to the surface emittance difference. The difference in convection heat flux is related to the velocity difference of the airflow above the sample, as shown in Supplementary Figure 13. Compared to the Cr case in which the surface-to-ambient radiation cannot interact with the above airflow, the radiation from the SE to the air (participating medium) heats the airflow and the velocity of the airflow is enhanced, as shown in the red dashed circle in Supplementary Figure 13(g). The enhanced airflow contributes to the reduction of the temperature for the fluid close to the solid-fluid boundary (indicated in the green dashed circle in Supplementary Figure 13(d)). Therefore, the convective heat transfer of the SE case is significantly larger than the Cr case, due to lower airflow temperature and higher airflow velocity.

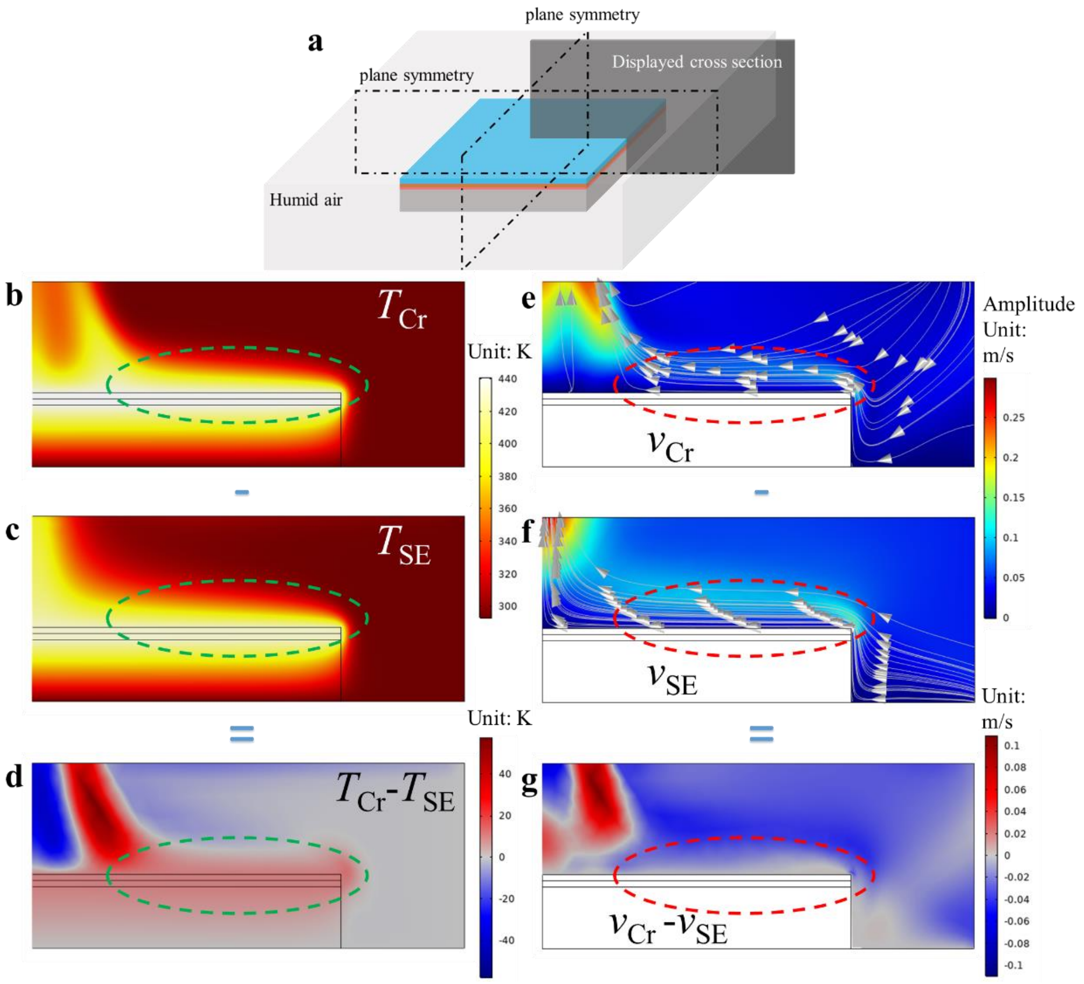

Supplementary Figure 13. Temperature and velocity field comparison between SE and Cr. (a) Schematic diagram of the conjugate heat transfer simulation. (b-c) The temperature field on the cross-section indicated in (a) for (b) Cr sample and (c) SE sample. (d) The temperature difference between Cr and SE cases. (e-f) The velocity amplitude field for (e) Cr sample and (f) SE sample. (g) The velocity difference amplitude between Cr and SE cases.

Supplementary note 10. Infrared camouflage demonstration of SE for larger temperature range

Infrared camouflage demonstration of SE for operating temperature ranging from 50 ℃ to 300 ℃ is shown in Supplementary Figure 14. At the sample temperature of 300 ℃, the radiation temperatures for the MWIR/LWIR band are 168.2 ℃/91.2 ℃, respectively. The radiation temperature is still significantly lower than the blackbody, indicated by an 83.9 %/77.1 % (7.94 dB/6.41 dB) reduction of IR signal intensity for MWIR/LWIR bands, respectively.

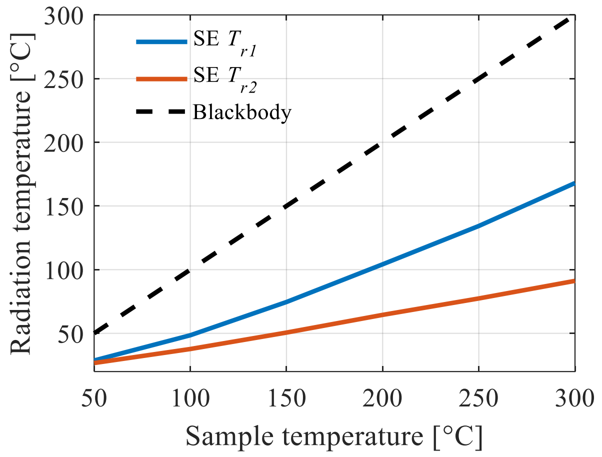

Supplementary Figure 14. Measured radiation temperature T_{r1}/T_{r2} in MWIR/LWIR band for SE at different sample temperatures.
